# Improvement of multiscale decomposition for space-based gravitational wave signal processing technology

**DOI:** 10.1371/journal.pone.0311213

**Published:** 2024-10-31

**Authors:** Qiuping Shen, Yunqing Liu, Dongpo Xu, Fei Yan, Siyuan Wu, Xin Chen

**Affiliations:** 1 School of Electronic Information Engineering, Changchun University of Science and Technology, Changchun, China; 2 Jilin Provincial Science and Technology Innovation Center of Intelligent Perception and Information Processing Changchun, Changchun, China; Xi’an Jiaotong University, CHINA

## Abstract

During the process of detecting gravitational waves in space, addressing noise issues caused by terrestrial vibrations, natural environmental changes, and the factors intrinsic to the detectors, this paper proposes a multiscale variational mode adaptive denoising algorithm based on momentum gradient descent. This algorithm integrates momentum factors and multiscale concepts into the variational mode algorithm to resolve the issue of multiple local optima encountered during operation, reduce oscillations in regions with large or unstable gradient changes, and improve convergence speed. Additionally, the algorithm combines the least mean squares algorithm to automatically adjust weights, thereby mitigating the impact of noise, addressing the issue of noise from multiple and random sources, effectively suppressing noise in the gravitational wave signal, and enhancing the quality and reliability of the gravitational wave signal. Experimental results demonstrate that this algorithm performs better than other algorithms in noise suppression, effectively reducing noise in gravitational wave signals and meeting the noise suppression requirements for space-based gravitational wave detection.

## Introduction

The discovery of gravitational waves has opened a new era in the field of astronomy [1]. These extremely faint perturbations, predicted by Einstein’s General Theory of Relativity [2], originate from extreme astrophysical events causing the curvature of spacetime, such as black hole mergers and neutron star collisions [3]. However, the detection of gravitational waves faces significant challenges, as these signals are extremely weak and susceptible to disturbances from Earth vibrations, variations in the natural environment, thermal noise and vibrations within the detectors, as well as interference from optical and electronic equipment [4]. The detectors require highly precise measurements of the propagation time of light to detect these subtle gravitational wave signals. Despite advancements, continuous efforts are needed to enhance the sensitivity of detectors and reduce the interference of noise with gravitational wave signals [5].

There is extensive research on the removal of noise from gravitational wave signals, with researchers dedicated to improving instrument design and denoising algorithms. Mours and collaborators studied thermal noise in gravitational wave antennas using higher-order transmission line modes. By designing specific mode properties of optical devices, they reduced the noise caused by detector materials and design, thereby decreasing the thermal noise from optical components and devices. However, this significantly increased the complexity of the interferometer, leading to higher difficulty and cost [6]. Nishizawa A and collaborators proposed using neutrons instead of light to address the issue of the displacement noise-free interferometer’s sensitivity band being too high for astrophysical gravitational wave sources. This approach aims to eliminate neutron displacement noise, but it demands high stability and precision of the experimental setup [7]. Tang and collaborators investigated a gravitational wave detection scheme using an atomic interferometer as an inertial sensor and reevaluated its structure through the concept of sensitivity functions. By adjusting the sensor spacing, they aimed to enhance detection sensitivity and performance while suppressing noise without affecting the gravitational wave signal. However, the actual detection environment can impact sensor performance and the effectiveness of the optimized spacing [8]. Rich Ormiston and collaborators used machine learning algorithms to reduce time series noise in gravitational wave detection caused by instrumental artifacts and environmental contamination. The signal-to-noise ratio of the injected signals was enhanced by approximately 21.6%, and the recovered parameters were consistent with the injected set. However, the model’s generalization ability may be limited when handling actual data with distributions different from the training data, especially in the presence of unknown noise [9].

Considering that the current research in the field of gravitational wave signal denoising mainly focuses on hardware aspects, we have decided to delve into algorithmic issues. The aim is to discover more effective methods for gravitational wave signal processing, hoping to provide new theoretical support and practical solutions for the accurate extraction and noise suppression of gravitational wave signals. This research is of significant importance in advancing the field of gravitational wave astrophysics.

This paper addresses the issue of slow convergence in gravitational wave signal processing by introducing a momentum gradient descent algorithm and the concept of multiscale decomposition to optimize the variational mode decomposition algorithm. These improvements accelerate convergence, prevent the search for the optimal solution from getting trapped in local optima, and enhance the decomposition accuracy and efficiency of the variational mode decomposition algorithm. In the context of large-scale data processing and real-time requirements, the incorporation of the least mean squares algorithm allows for automatic parameter adjustment under complex and randomly varying noise conditions, reducing errors and enhancing stability and robustness. First, based on the characteristics of the gravitational wave signal, momentum factors are introduced, and the optimal parameters for decomposition are selected to perform variational mode decomposition on the gravitational wave signal [10–12]. Then, the least mean squares algorithm is used to denoise each mode component [13–15]. Finally, the denoised mode components are reconstructed to obtain the denoised gravitational wave signal. Experiments demonstrate that this denoising algorithm improves convergence speed, enables real-time adaptive parameter adjustment for denoising, significantly enhances the signal-to-noise ratio of gravitational wave signals, and improves the accuracy and reliability of signal extraction, showing excellent performance in noise reduction.

## Materials and methods

### Gravitational wave signal analysis

Gravitational waves are spacetime disturbances caused by extreme astrophysical events, originating from the acceleration changes of massive objects in the universe [16, 17]. Their amplitude is extremely weak, possibly reaching variations at the nanometer or even picometer levels when arriving at Earth, making them challenging to detect. Highly sensitive scientific instruments such as laser interferometers are required for detection. However, gravitational wave detectors face various sources of noise interference, including external environmental disturbances, internal vibrations, and gravitational gradient noise within the detectors [18]. These interference factors can mask gravitational wave signals, making them more challenging to be accurately detected and identified. The noise in gravitational waves is determined by multiple factors, leading to the representation of gravitational waves as a combination of signal and noise components.

The gravitational wave signal was calculated by using (1):

$$\begin{array}{c} f(t)=u(t)+z(t) \end{array}$$ (1)

where, *f*(*t*) represents the gravitational wave signal, and *z*(*t*) represents complex Gaussian white noise. *z*(*t*) represents the noise of the gravitational wave signal.

The noise encountered during gravitational wave detection is primarily divided into three categories: front-end optical noise, analog circuit noise, and digital circuit noise.

Front-end optical noise includes photodetector noise, Doppler shift noise, jitter path coupling noise and quantum shot noise. This type of noise directly affects the conversion efficiency of optoelectronic signals, reduces the sensitivity of the detector, and increases the error in signal detection. Taking quantum shot noise as an example, it is detection noise caused by statistical fluctuations in the number of photons. This means there is a slight quantum fluctuation limit on the stability of the incident laser intensity. The equation is as follows:

$$\begin{array}{c} (\delta x_{s}{)}^{2}=\frac{hc\lambda △f}{8\pi^{2}P}+\frac{8Phv△f}{m^{2}\omega^{4}c^{4}} \end{array}$$ (2)

where, *δx*_*s*_ represents equivalent displacement noise, *h* represents planck constant, *c* represents speed of light, λ represents wavelength of the laser in vacuum, *P* represents total optical power of interference laser, △*f* represents observation bandwidth, *m* represents check the inertial mass of the mass, *ω* represents signal angular frequency.

Analog circuit noise includes amplifier thermal noise, four-quadrant photodetector noise and low-frequency thermal drift noise, and others. This type of noise superimposes on the gravitational wave signal, not only increasing the base noise and reducing the signal-to-noise ratio, but also affecting signal amplification and transmission. Additionally, it can lead to signal aliasing and distortion, impacting the accuracy of position and intensity measurements. Taking four-quadrant photodetector noise as an example, due to the different charge quantities between quadrants, the photodiodes generate induced capacitance with air and the depletion layer acting as dielectrics. This results in adjacent quadrant signals inducing local crosstalk noise. The equation is as follows:

$$\begin{array}{c} \delta \varphi_{ct}=\delta (\frac{\alpha \cdot A_{S_{n}}}{A_{S_{0}}})\cdot \text{cos}\langle S_{0},S_{n}\rangle \end{array}$$ (3)

where, *S*_0_ represents local signal, *S*_*n*_ represents crosstalk signal, *α* represents photodiode isolation, $\delta A_{S_{n}}$ represents crosstalk signal amplitude noise, $\delta A_{S_{0}}$ represents local signal amplitude noise.

Digital circuit noise includes sampling time jitter noise, quantization noise, phase-locked loop noise, and others. This type of noise can cause signal distortion and spectral broadening, affecting the phase stability and frequency locking accuracy of the signal. As a result, it leads to inaccurate and unsynchronized phase measurements. Taking sampling time jitter noise as an example, during the sampling process, noise is introduced due to the offset in sampling time caused by clock signal jitter or non-idealities in the sampling circuit. The equation is as follows:

$$\begin{array}{c} \sigma_{j}^{2}=\frac{1}{12}△t^{2}△f_{s}^{2} \end{array}$$ (4)

where, *σ*_*j*_ represents standard deviation of sampling jitter noise, △*t* represents the offset of sampling time, △*f*_*s*_ represents the offset of the sampling rate.

Gravitational wave signals have extremely low amplitudes but a broad frequency range, spanning from millihertz to kilohertz and even higher. Consequently, the signal-to-noise ratio requirements for detection equipment are very stringent. It is crucial to effectively suppress various noise sources, such as thermal noise, environmental noise, and instrumental noise, to ensure the reliable detection of gravitational wave signals. Therefore, reducing noise interference is crucial in the process of gravitational wave detection. By minimizing noise interference, the signal-to-noise ratio and quality of gravitational wave signals can be improved, leading to more accurate measurements of various parameters of gravitational wave events. This provides better data support for the development of gravitational wave astronomy, enabling scientists to detect and interpret gravitational wave data more precisely, and thus gain a deeper understanding of the universe.

### Adaptive variational mode decomposition denoising algorithm optimized by momentum gradient descent

#### Variational mode decomposition

Gravitational waves propagate at the speed of light, but their amplitude rapidly decays with increasing distance. When gravitational waves reach Earth, their amplitude has been significantly attenuated, typically reaching levels on the order of nanometers or picometers. This extreme weakness makes the detection and capture of gravitational waves extremely challenging. Variational mode decomposition (VMD) [19, 20] is a signal processing technique that decomposes complex signals into multiple local frequency modes. It can decompose gravitational wave signals at smaller scales, suppressing noise and reducing interference with gravitational wave signals at smaller local frequency modes, thereby enhancing the accuracy of detected gravitational wave signals.

Variational mode decomposition decomposes gravitational wave signals into intrinsic mode function (IMF) with frequency and amplitude modulation characteristics and sparse properties [21–23]. It assumes that each order mode is compactly centered around the central frequency and estimates the bandwidth by the *L*^2^ norm of the corresponding demodulated signal. The gravitational wave signal f(t) is decomposed into *m* IMF components, yielding the constrained variational model:

$$\begin{array}{c} \underset{\left\{u_{m}\}\{\omega_{m}\right\}}{min}\{\sum_{i=1}^{m}\|\begin{array}{c} \partial_{t}[(\delta \left(t\right)+\frac{j}{\pi t})*u_{m}\left(t\right)e^{-j\omega_{m}t}] \end{array}{\|}_{2}^{2}\} \end{array}$$ (5)

$$\begin{array}{c} s.t.\sum_{i=1}^{m}u_{m}=f \end{array}$$ (6)

where, *u*_*m*_ represents the *m*-th decomposed mode component, and *ω*_*m*_ denotes the central frequency of the *m*-th mode component.

Using the alternating direction method of multipliers for solution, the constrained optimization problem is transformed into an unconstrained variational problem. Introducing a quadratic penalty factor *ρ* and Lagrange multiplier λ, we obtain the augmented Lagrangian function, expressed as:

$$\begin{array}{c} \begin{array}{cc} & L(u_{m},w_{m},\lambda )=\rho \sum_{i=1}^{m}\|\partial t[(\delta (t)+\frac{j}{\pi t})*u_{m}(t)]e^{-jw_{m}t}{\|}_{2}^{2}\\ & +\lambda^{H}(f(t)-\sum_{i=1}^{m}u_{m}(t))+\|f(t)-\sum_{i=1}^{m}u_{m}(t){\|}_{2}^{2} \end{array} \end{array}$$ (7)

Using the following equations to solve and update *u*_*m*_, *ω*_*m*_, and λ:

$$\begin{array}{c} {\hat{u}}_{m}^{k+1}(\omega )=\frac{\varphi }{\left[1+2\rho (\omega -\omega_{m}^{k}{)}^{2}\right]} \end{array}$$ (8)

$$\begin{array}{c} \omega_{m}^{k+1}(\omega )=\frac{\int_{0}^{\infty }\omega |{\hat{u}}_{m}^{k+1}(\omega ){|}^{2}d\omega }{\int_{0}^{\infty }|{\hat{u}}_{m}^{k+1}(\omega ){|}^{2}d\omega } \end{array}$$ (9)

$$\begin{array}{c} {\hat{\lambda }}^{k+1}(\omega )={\hat{\lambda }}^{k}(\omega )+\zeta (\hat{f}(\omega )-\sum_{i=1}^{m}{\hat{u}}_{m}^{k+1}) \end{array}$$ (10)

$$\begin{array}{c} \varphi =[f(\omega )-\sum_{i=1,i‡m}^{M}{\hat{u}}_{i}^{k+1}(\omega )+\frac{{\hat{\lambda }}^{k}(\omega )}{2}] \end{array}$$ (11)

where, *ζ* represents the noise tolerance. When the signal contains strong noise, setting *ζ* = 0.

The variational mode decomposition algorithm decomposes each mode into different frequencies based on their central frequency and bandwidth, making the separation between noise and signal clearer. This reduces the impact of noise and improves the signal-to-noise ratio. Variational mode decomposition not only enhances the detection capability of gravitational wave signals but also improves the accuracy and reliability of the analysis. This enables gravitational wave detectors to more precisely capture weak gravitational wave signals, providing a clearer and more reliable signal foundation for subsequent scientific research and data analysis.

#### Improved variational mode adaptive denoising algorithm

The variational mode decomposition algorithm decomposes weak gravitational wave signals into multiple local frequency modes, involving a non-convex optimization problem that may have multiple local optima. Given that gravitational wave signals are weak and embedded in substantial noise, finding the global optimum for the variational mode decomposition is challenging. Therefore, this paper introduces the gradient descent algorithm, which iteratively optimizes and gradually approaches the optimal solution. This approach helps avoid getting trapped in local optima to some extent, achieving better decomposition results and accelerating convergence. As a result, the optimization process reaches a stable state more quickly, improving the computational efficiency of the algorithm.

The gradient indicates the direction of the steepest descent. Updating the modes and central frequencies in the opposite direction of the gradient allows for finding the global optimum solution,

$$\begin{array}{c} {\hat{u}}_{m}^{\prime }(\omega )={\hat{u}}_{m}^{k}(\omega )-\alpha ▽L \end{array}$$ (12)

$$\begin{array}{c} \omega_{m}^{\prime }(\omega )=\omega_{m}^{k}(\omega )-\alpha ▽L \end{array}$$ (13)

where, *α* represents learning rate.

However, as the gradient descent method slows down when approaching local minima, momentum is introduced. This includes assigning larger weights to gradients closer to the current iteration and smaller weights to those further away. This weight distribution helps reduce oscillations in regions with large or unstable gradient changes, allowing for smoother convergence towards the optimal solution. Additionally, it enhances the computational efficiency of the optimization process by reducing unnecessary parameter updates and direction changes, thus accelerating convergence speed. The momentum term is given by:

$$\begin{array}{c} v_{k}=\beta v_{k-1}+(1-\beta )▽L_{k-1} \end{array}$$ (14)

where, *β* represents momentum coefficient. Therefore, the modal and central frequency updates for the *k* + 1 iteration are given by:

$$\begin{array}{c} {\hat{u}}_{m}^{k+1}(\omega )={\hat{u}}_{m}^{\prime }(\omega )-\alpha v_{k} \end{array}$$ (15)

$$\begin{array}{c} \omega_{m}^{k+1}(\omega )=\omega_{m}^{\prime }(\omega )-\alpha v_{k} \end{array}$$ (16)

In addition, this paper incorporates a multiscale decomposition approach to optimize regularization weights, aiming to strike the optimal balance between preventing overfitting and maintaining high prediction accuracy. Considering the influence of noise on gravitational wave signals over time, this approach extracts noise at smaller scales. Therefore, the gravitational wave signal is decomposed at scale *ρ* ⋅ 2^−1^:

$$\begin{array}{c} \begin{array}{cc} & L(u_{m},w_{m},\lambda )=\rho \cdot 2^{-1}\sum_{i=1}^{m}\|\partial t[(\delta (t)+\frac{j}{\pi t})*u_{m}(t)]e^{-jw_{m}t}{\|}_{2}^{2}\\ & +\lambda^{H}(f(t)-\sum_{i=1}^{m}u_{m}(t))+\|f(t)-\sum_{i=1}^{m}u_{m}(t){\|}_{2}^{2} \end{array} \end{array}$$ (17)

The penalty factor directly affects the results of the gravitational wave signal decomposition. If it is too large, it will restrict the bandwidth of each IMF component, resulting in a narrower frequency range and the loss of some information, sacrificing the optimization of the objective function. If it is too small, it will allow a wider frequency range, causing mode mixing and sacrificing the purity of each IMF component, affecting the accuracy and precision of the decomposition. Therefore, decomposing at the scale of *ρ* ⋅ 2^−*k*^:

$$\begin{array}{c} \begin{array}{cc} & L(u_{m},w_{m},\lambda )=\rho \cdot 2^{-k}\sum_{i=1}^{m}\|\partial t[(\delta (t)+\frac{j}{\pi t})*u_{m}(t)]e^{-jw_{m}t}{\|}_{2}^{2}\\ & +\lambda^{H}(f(t)-\sum_{i=1}^{m}u_{m}(t))+\|f(t)-\sum_{i=1}^{m}u_{m}(t){\|}_{2}^{2} \end{array} \end{array}$$ (18)

As the decomposition scale continues to refine, useful detailed information is continually extracted, simultaneously separating useful information from noise, thus improving the accuracy of the gravitational wave signal. This improvement accelerates the algorithm’s speed, enhances the signal-to-noise ratio, and is of significant importance for the advancement of gravitational wave astronomy. It enables scientists to better understand gravitational wave phenomena in the universe.

While the variational mode decomposition effectively decomposes gravitational wave signals into multiple intrinsic mode functions, it does not guarantee that each IMF exclusively contains clear gravitational wave signals; some IMFs may include noise or other interference components. This is particularly challenging in complex noise environments where eliminating all noise completely is difficult. Therefore, introducing the least mean squares (LMS) algorithm can further denoise each IMF, reducing noise levels and enhancing the signal-to-noise ratio, clarity, and accuracy of the signals.

**Fig 1:**
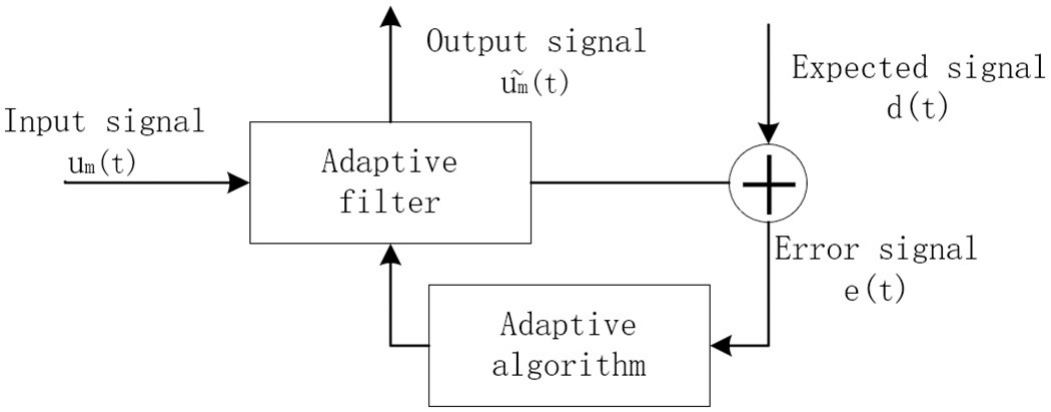
Structure diagram of the adaptive filter.

Gravitational wave noise typically appears in a random manner, and the amplitude and characteristics of gravitational wave signals may vary over time and in different environments, making it challenging to predict and capture gravitational wave signals. The least mean squares algorithm is an adaptive filtering technique capable of automatically adjusting weights based on the characteristics of gravitational wave signals to reduce the impact of noise. It does not require prior knowledge of the statistical properties of the noise. Instead, through iterative learning of the features of gravitational wave signals, the algorithm makes incremental adjustments at each step to minimize the mean square value of prediction errors. Therefore, the least mean squares algorithm can effectively suppress noise in gravitational wave signals without prior knowledge of noise characteristics, enhancing the quality and reliability of gravitational wave signals [24–26]. The structure of an adaptive filter is illustrated in Fig 1.

Utilizing the least mean squares algorithm, adaptive filtering is applied to the gravitational wave signal *u*_*j*_ (*t*) to further mitigate noise interference. In this process, the gravitational wave signal is defined as the target signal, while noise is considered as the interfering reference signal. By continuously monitoring real-time signal inputs, calculating the error between the target signal and the reference signal, and dynamically updating the filter weights based on the rules of the least mean squares algorithm, the system adapts to minimize the error [27–29]. This adaptive process enables the filter to effectively suppress noise components, resulting in a clearer output signal and providing more accurate results for the detection of gravitational wave signals.

The output of the filter is given by:

$$\begin{array}{c} \tilde{u}(t)=W^{H}(t)u_{m}(t)=\sum_{i=0}^{N-1}w_{i}(t)u_{m}(t-i) \end{array}$$ (19)

where, *u*_*m*_ (*t*) represents the input vector, *H* represents the transpose, *W* (*t*) represents the weight coefficient vector, and *N* represents the filter order.

$$\begin{array}{c} u_{m}(t)=[u_{m}(t),u_{m}(t-1),\ldots ,u_{m}(t-N+1){]}^{H} \end{array}$$ (20)

$$\begin{array}{c} W(t)=[w_{0}(t),w_{1}(t-1),\ldots ,w_{N-1}(t){]}^{H} \end{array}$$ (21)

The estimation error signal is given by:

$$\begin{array}{c} e(t)=d(t)-\tilde{u}(t) \end{array}$$ (22)

The weight vector updating equation is given by:

$$\begin{array}{c} W(t+1)=W(t)+2\mu u_{m}(t)e(t) \end{array}$$ (23)

where, *μ* represents the step size factor. To ensure the convergence of the LMS algorithm, the step size factor needs to satisfy the condition $0<\mu <\frac{1}{\lambda_{max}}$, where λ_*max*_ represents the maximum eigenvalue of the autocorrelation matrix of the input signal.

#### Reconstruction of gravitational wave signals

Due to the weak nature of gravitational wave amplitudes, it is necessary to reconstruct the modes after noise removal, distinguishing genuine gravitational wave components from noise to enhance signal accuracy. The reconstruction process involves assembling the small-scale signal components processed through enhanced variational mode decomposition and least mean square algorithm into the denoised gravitational wave signal. Summing all processed mode components,

$$\begin{array}{c} \tilde{u}(t)=\tilde{u_{0}}(t)+\cdots +\tilde{u_{j}}(t)=\sum_{j=0}^{k}{\tilde{u}}_{j}(t) \end{array}$$ (24)

where, $\tilde{u_{j}}(t)(j=0,1,\cdots ,k)$ represents the denoised mode components, $\tilde{u}(t)$ represents the reconstructed gravitational wave signal.

This process ensures that all frequency components of the gravitational wave signal are accurately extracted and restored, resulting in a cleaner reconstructed signal, thereby improving the quality and accuracy of the gravitational wave signal.

#### Overall algorithm flow

First, acquire the information collected by the detectors as the raw signal. Next, utilize momentum gradients and multiscale concepts to optimize variational modes, avoiding multiple local optima during operation and achieving fast-converging optimal decomposition. Then, decompose the raw signal using the variational mode decomposition to obtain modes of different frequencies. Apply least mean squares suppression to these noisy frequency modes, adaptively removing noise contained in the gravitational wave signal. Finally, recombine the denoised modes to obtain a cleaner gravitational wave signal. The overall algorithm flow is shown in Table 1.

**Table 1:** Detailed procedure of the adaptive denoising algorithm for space-based gravitational wave signals based on multi-scale decomposition.

Algorithm:Adaptive Denoising Algorithm for Space-based Gravitational Wave Signals based on Multi-scale Decomposition
Input:Original signal with noise *f*, penalty term coefficient *ρ*
1.Initialize: λ, {*u*_*m*_}, {*ω*_*m*_}, *k* = 1, *n* ← 0
2.while: $\sum_{m=1}^{M}(\|{\hat{u}}_{m}^{k+1}-{\hat{u}}_{m}^{k}{\|}_{2}^{2})/(\|{\hat{u}}_{m}^{k}{\|}_{2}^{2})<\eta$
3.update ${\hat{\lambda }}^{k+1},{\hat{u}}_{m}^{k+1},\omega_{m}^{k+1}$ by using Eqs (10), (12) and (13)
4.initialize:*α*, *β*,*k* = *k* + 1
5.*v*_*k*_ = *βv*_*k*−1_ + (1 − *β*) ▽ *L*_*k*−1_
6.update ${\hat{u}}_{m}^{k+1}(\omega ),\omega_{m}^{k+1}(\omega )$ by using Eqs (15) and (16)
7.*ρ*_*k*_ = *ρ* ⋅ 2^−*k*^
8.update *L* by using Eq (18)
9.end while
10.extracting the signal *u*_*j*_(*j* = 0, 1, …, *k*)
11.update *W*(*t*) by using Eq (23)
12.filtering the *u*_*j*_ signal separately by using Eqs (20), (21) and (22)
13.calculate ${\tilde{u}}_{j}$ by using Eq (19)
14.reconstruct each signal components for denoising ${\tilde{u}}_{j}$
Output:Decomposed gravitational wave signal $\tilde{u}=\sum_{i=0}^{k}{\tilde{u}}_{j}$

## Experiments and results

### Measuring system

**Fig 2:**
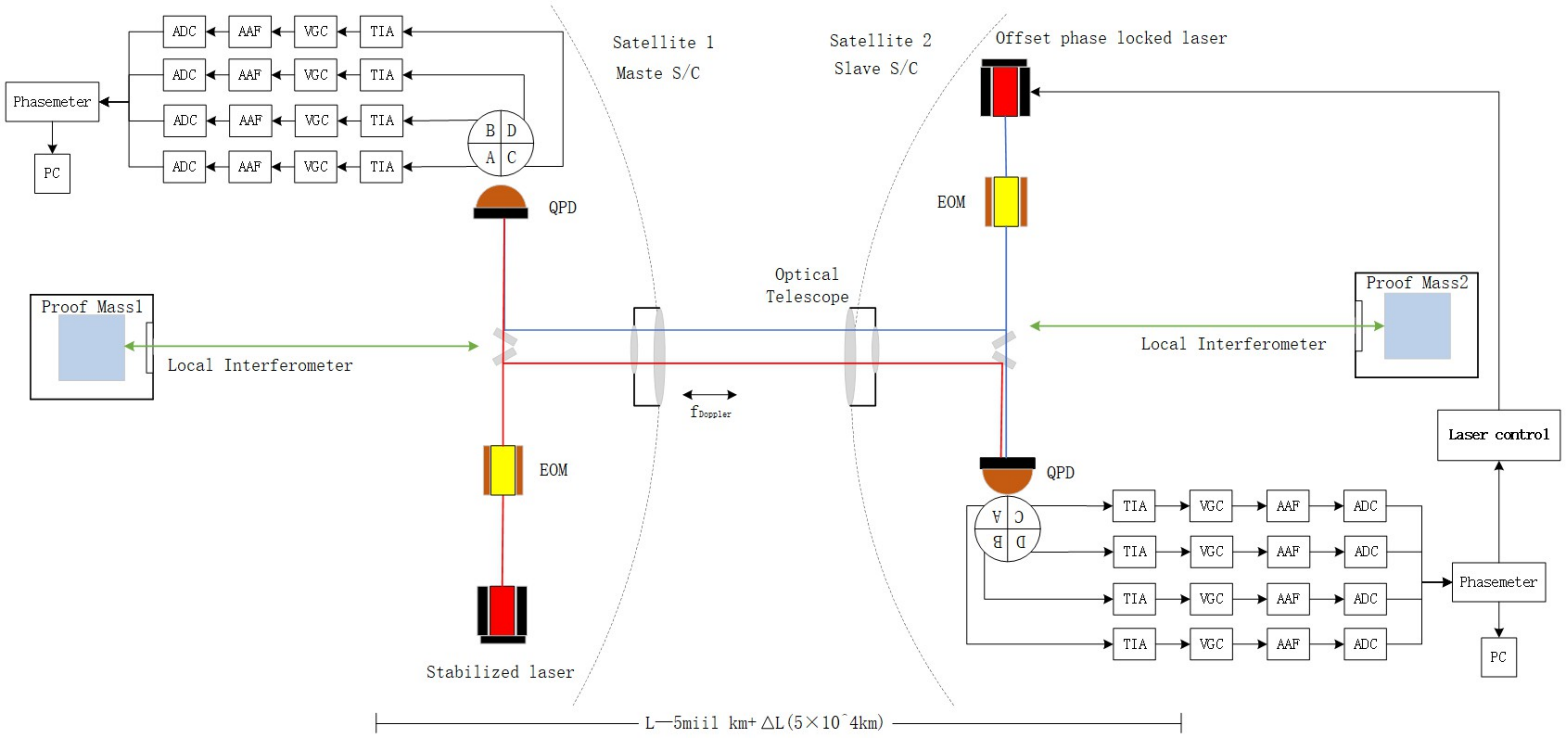
Schematic of the principle of laser heterodyne interferometry for space-based gravitational wave detection.

The principle of laser heterodyne interferometry for space-based gravitational wave detection is illustrated in Fig 2. The spaceborne laser interferometer antenna consists of three satellites, each separated by 5 million kilometers and connected via three bidirectional laser links. Paired test masses from different satellites serve as the end mirrors of the interferometer, with the interferometric measurement system monitoring optical path fluctuations caused by gravitational waves. The two satellites carry separate laser sources, one is a stable laser and the other is a biased phase-locked laser. Satellite 2 receives laser light emitted from Satellite 1, which is reflected off test mass 2 on Satellite 2 and then interferes with laser 2. This interference signal sequentially passes through a quadrant photodetector (QPD), a transimpedance amplifier (TIA), a variable gain controller (VGC), an anti-aliasing filter (AAF), and an analog-to-digital converter (ADC), converting it into an electrical signal. The phase meter reads out the phase difference between laser 2 and the received laser. Using weak light phase-locking, laser 2 and the received laser are locked in differential frequency phase, allowing laser 2 to carry the phase information of the received laser. The phase-locked laser 2 is then transmitted back to Satellite 1, where it reflects off test mass 1 and interferes with laser 1. The heterodyne interference signal thus contains information on the distance variation between the test masses of the two satellites. By measuring the phase change of the laser interference signal on Satellite 1 with a phase meter, the distance variation between the test masses caused by gravitational waves can be inferred [30].

### Measured data processing and analysis

**Fig 3:**
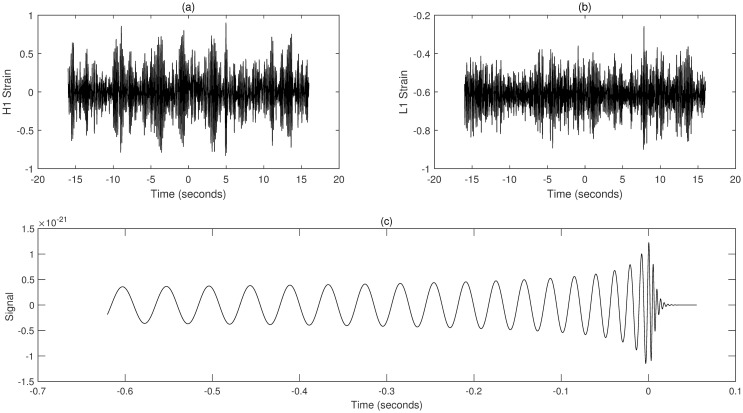
Schematic of spatial gravitational wave signal detection. (a) Gravitational wave strain signal recorded by the H1 detector. (b) Gravitational wave strain signal recorded by the L1 detector. (c) Detected gravitational wave signal in actual detection.

The data used in this study are all sourced from the official website of the Laser Interferometer Gravitational-Wave Observatory (LIGO). The detectors located in Hanford, Washington (H1), and Livingston, Louisiana (L1), together form the LIGO observatory. These detectors are used to measure spacetime strains caused by passing gravitational waves. By reading data from both the H1 and L1 detectors, gravitational wave signals can be identified when near-simultaneous signals with consistent waveforms are detected by both detectors. Fig 3 shows the gravitational wave strain signals recorded by the H1 and L1 detectors and the detected gravitational wave signal. This signal is used as a reference signal and is compared with the actual detected signal to help identify and validate the characteristics of gravitational wave events.

**Fig 4:**
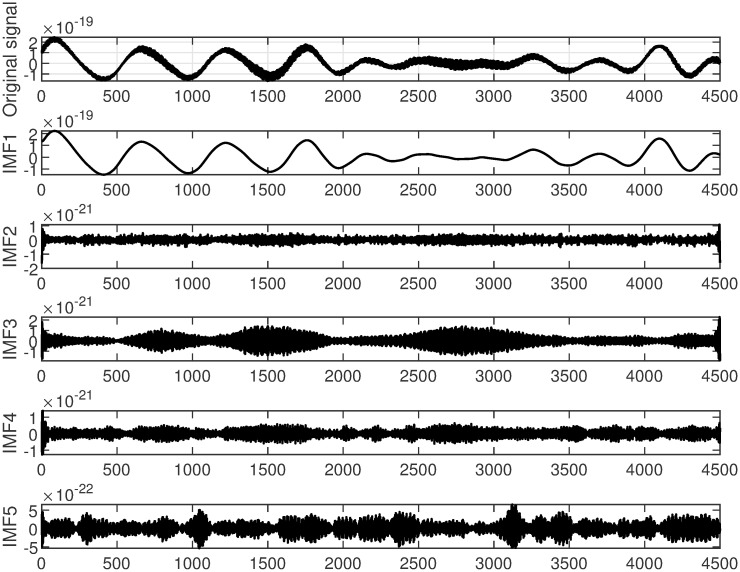
VMD decomposition of spatial gravitational wave signal diagram.

**Fig 5:**
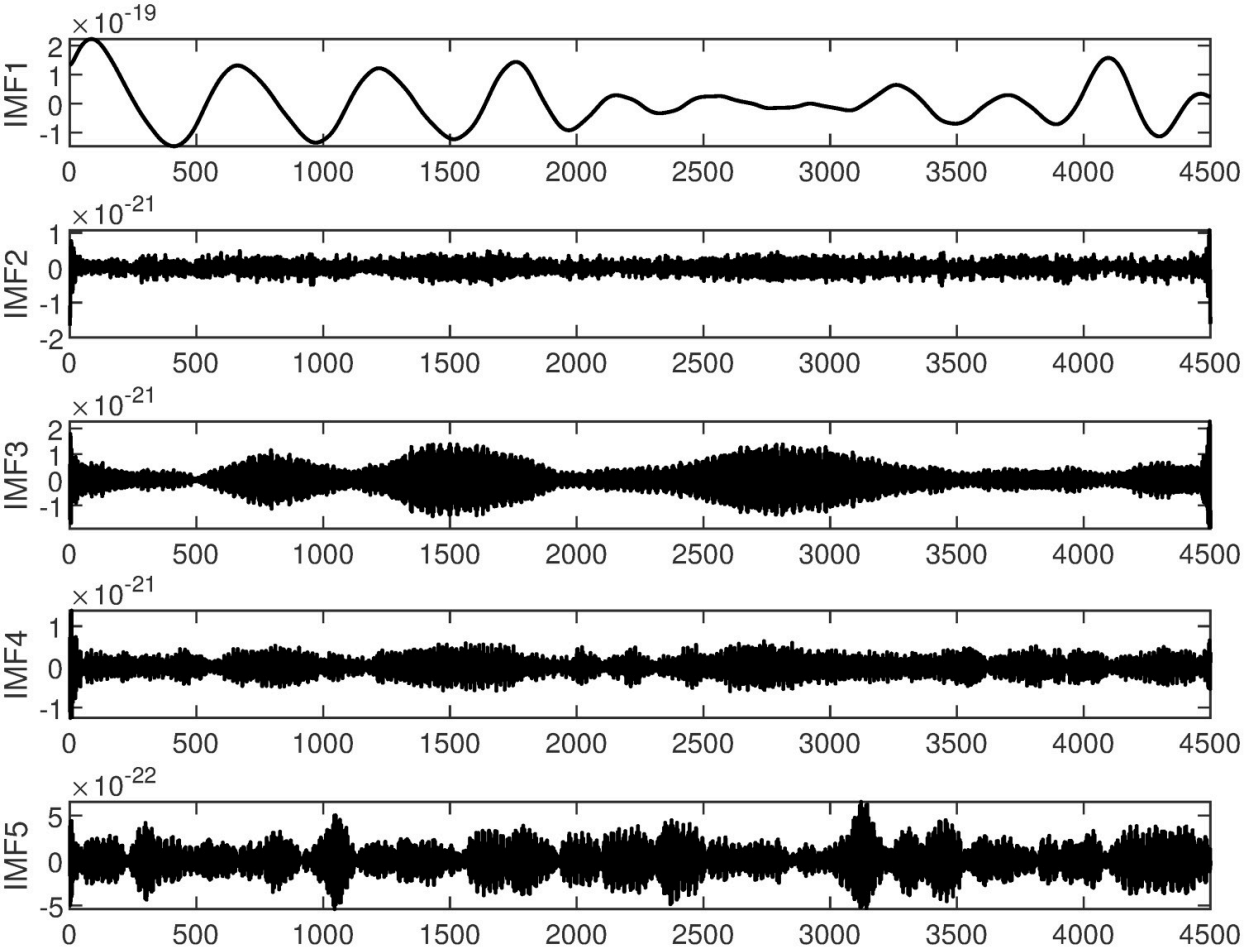
LMS suppression modal component diagram.

**Fig 6:**
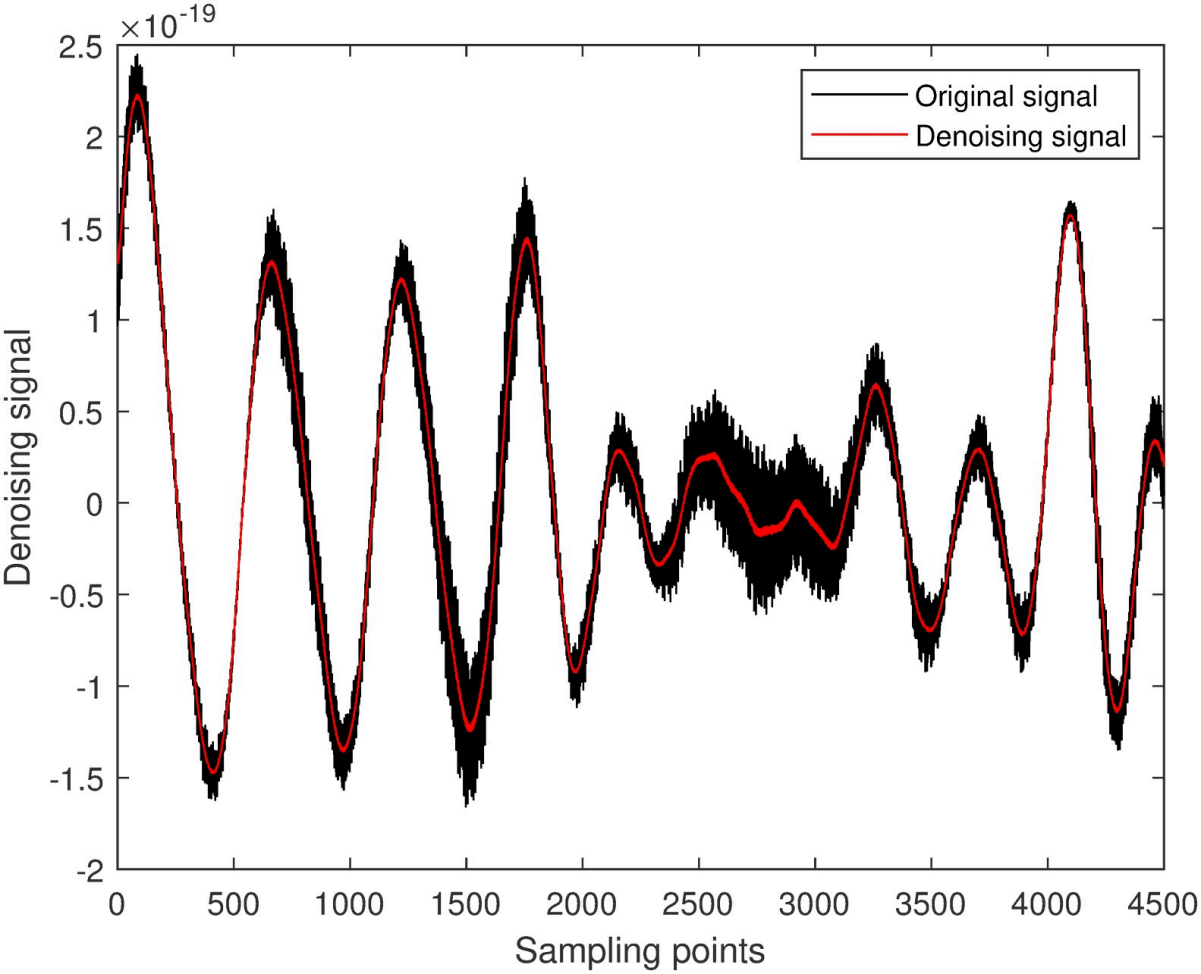
Space gravitational wave signal denoising diagram.

In this study, GW150914 is taken as an example. GW150914 originated from the merger of two black holes and was detected by the Laser Interferometer Gravitational-Wave Observatory (LIGO) between September 12 and October 20, 2015. It was the first direct observation of gravitational waves, confirming a significant prediction of Einstein’s theory of general relativity. The multiscale decomposition algorithm for gravitational wave signals involves three processing steps. Firstly, the improved variational mode decomposition algorithm is used to decompose the signal into different local frequency modes. The choice of decomposition scale is crucial during variational mode decomposition denoising processing. A larger decomposition scale results in the signal being decomposed into more sub-signals, allowing for finer denoising that preserves more signal characteristics. However, if the decomposition scale is too large, it may introduce additional noise and aliasing, leading to poor denoising effects and potentially larger errors. On the other hand, too small of a decomposition scale can disrupt or lose signal characteristics, thereby affecting denoising effectiveness. Therefore, selecting an appropriate decomposition scale is crucial. The gravitational wave signal is extremely weak and heavily disturbed by noise during detection. Therefore, the gravitational wave signal is decomposed into 5 layers, as shown in Fig 4. The first component is the raw gravitational wave signal, which is the initial signal without variational mode decomposition processing. The other components represent modal components obtained through variational mode decomposition decomposition, with each layer representing local modes of different frequencies and bandwidths. These components encapsulate various frequency-domain components of the original gravitational wave signal. Next, the least mean square adaptive algorithm is applied to suppress noise in each local mode by continuously adjusting the filter weights, gradually reducing the noise impact on the signal, as shown in Fig 5. Finally, the optimal output of each IMF optimized by the least mean square algorithmm is combined. At this stage, each local mode has undergone noise suppression and optimization, resulting in a more precise and cleaner gravitational wave signal, as illustrated in Fig 6.

**Fig 7:**
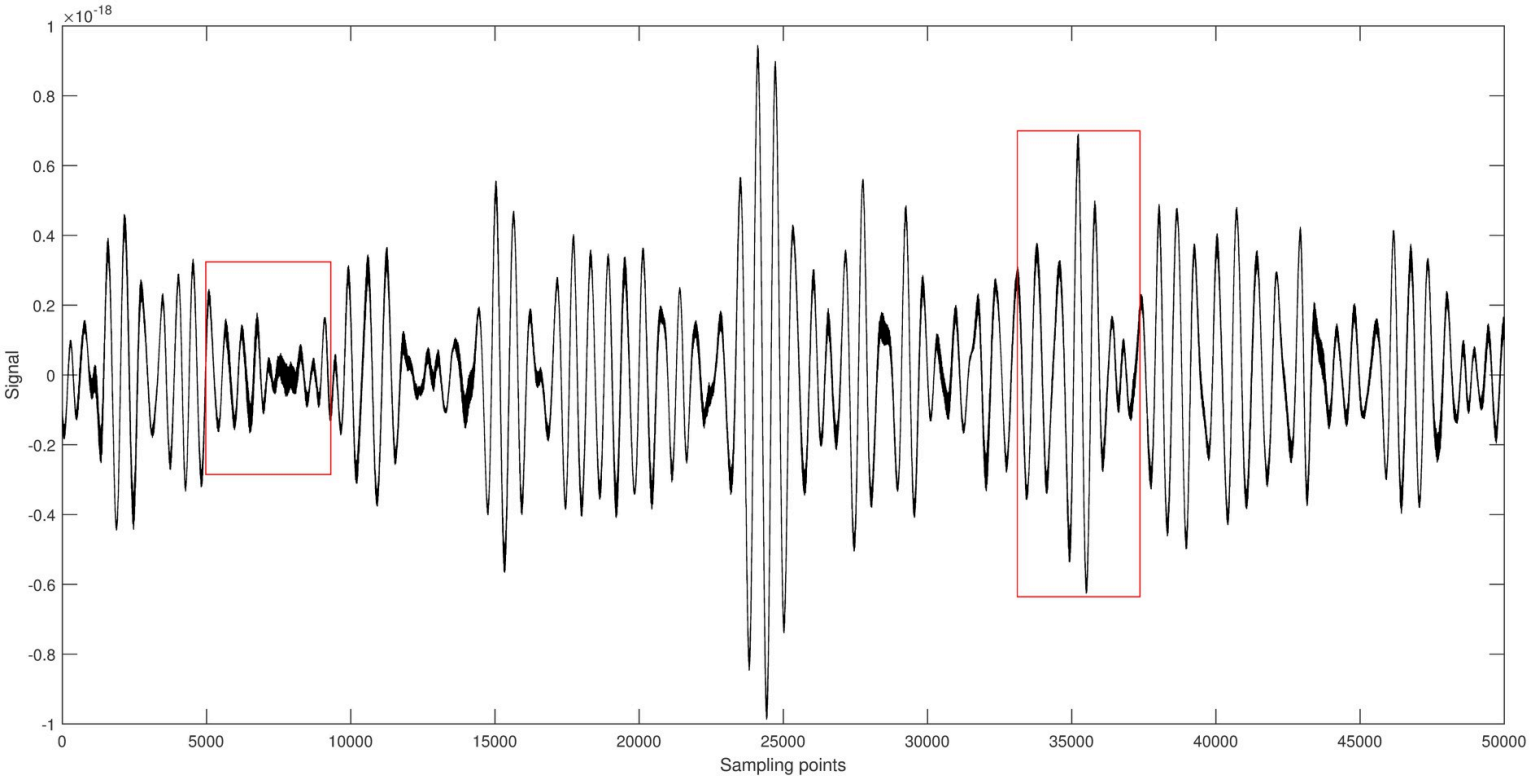
Space gravitational wave signal GW150914 diagram.

**Fig 8:**
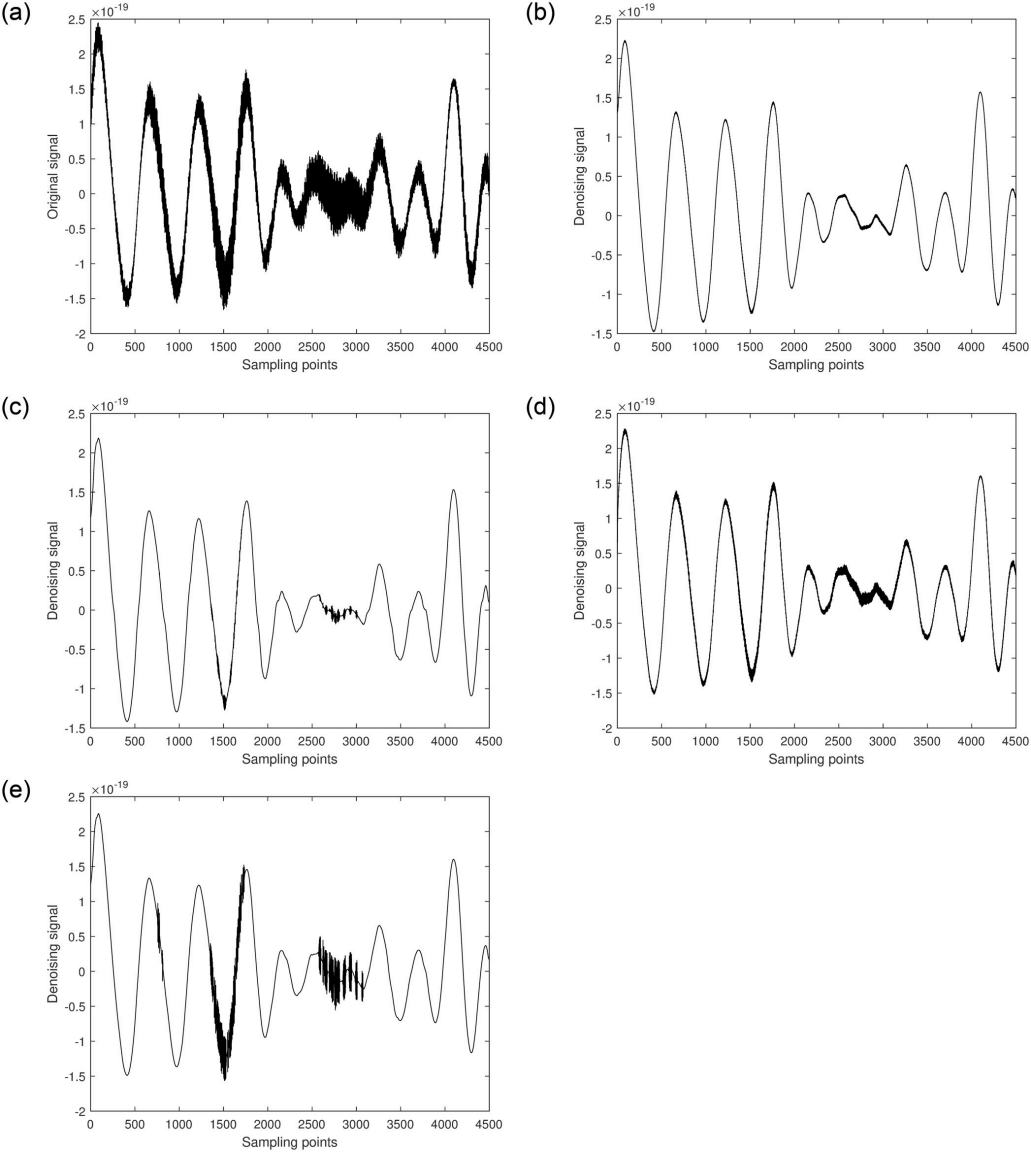
a. [5001-9500] part of the original signal. b. Multiscale decomposition algorithm for [5001-9500] part denoising processing. c. Kalman filter for [5001-9500] part denoising processing. d. Wavelet soft threshold for [5001-9500] part denoising processing. e. Wavelet hard threshold for [5001-9500] part denoising processing.

**Fig 9:**
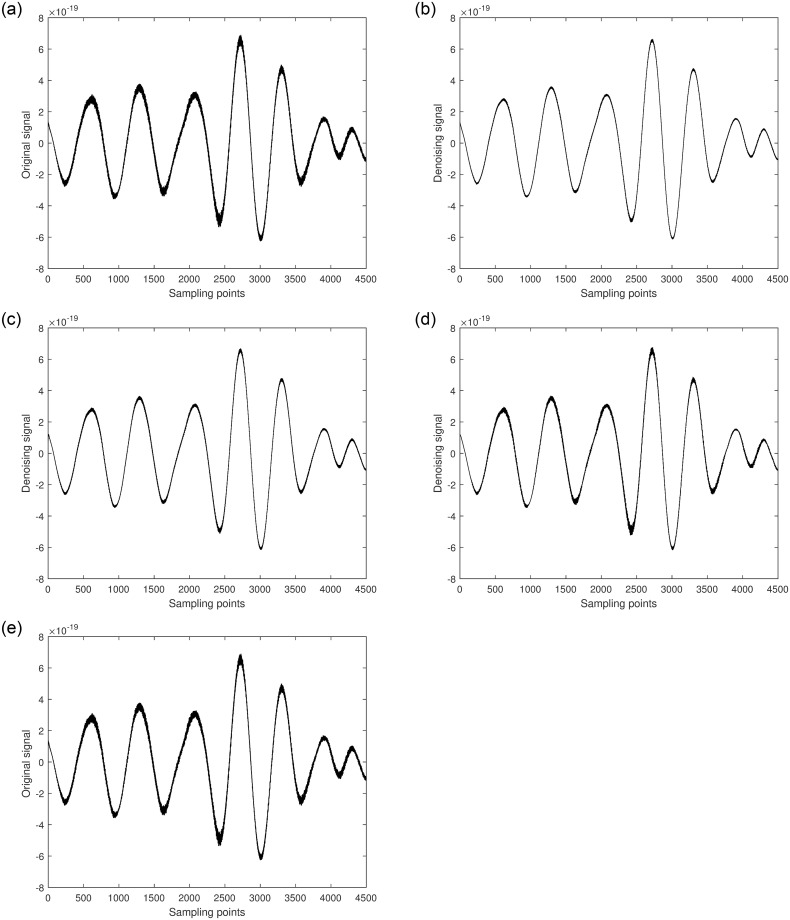
a. [32501-37000] part of the original signal. b. Multiscale decomposition algorithm for [32501-37000] part denoising processing. c. Kalman filter for [32501-37000] part denoising processing. d. Wavelet soft threshold for [32501-37000] part denoising processing. e. Wavelet hard threshold for [32501-37000] part denoising processing.

Fig 7 shows the original signal of GW150914. It is randomly segmented into two parts [5001-9500] and [32501-37000], each part having a length of 4500. Different algorithms are applied to denoise each segment, and the results are shown in Figs 8 and 9.

From the figures, it can be seen that compared to the original gravitational wave signal, the quality of the segments [5001-9500] and [32501-37000] has improved to varying degrees after denoising with the Kalman filter [31]. Although the Kalman filter can significantly recover the gravitational wave signal, its capability to handle non-Gaussian noise is limited, particularly for smaller amplitude noise. After denoising with wavelet thresholding [32], the quality of the signal segments [5001-9500] and [32501-37000] improved significantly. Among them, the wavelet thresholding method using the hard threshold function had the worst denoising effect. It only removed most of the noise but almost failed to outline the effective components of smaller amplitude signals. Most of the effective signal components remained mixed with noise, resulting in some artifacts. The wavelet thresholding method using the soft threshold function achieved better denoising results. It not only significantly removed most of the noise but also effectively filtered out noise from higher amplitude signals, making the waveform smoother. However, some effective local waveforms still did not show significant denoising improvement. The multiscale decomposition denoising method proposed in this paper achieved the best denoising results. It effectively removed most of the noise from the original gravitational wave signal and successfully delineated almost all the effective signal components, resulting in a smoother waveform. This demonstrates the reliability and superiority of the proposed multi-scale decomposition denoising algorithm.

In order to better assess the denoising effectiveness of various algorithms on gravitational wave signals, we adopt the common criteria of calculating the Signal to Noise Ratio (SNR) and Peak Signal to Noise Ratio (PSNR). Additionally, for a more objective and accurate evaluation of the denoising performance, the Mean Squared Error (MSE) algorithm is employed. SNR, PSNR, and MSE have long been conventional methods for measuring the effectiveness of noise reduction algorithms. Their definitions are as follows:

$$\begin{array}{c} SNR=10\;\text{lg}\{\sum_{n=0}^{N-1}x^{2}(n)/\sum_{n=0}^{N-1}[x^{2}(n)-y^{2}(n)]\} \end{array}$$ (25)

$$\begin{array}{c} MSE=\frac{1}{L}\sum_{i=1}^{n}[x(n)-y(n){]}^{2} \end{array}$$ (26)

$$\begin{array}{c} PSNR=10\;\text{lg}({MAX}^{2}/MSE) \end{array}$$ (27)

where, *x*(*n*) represents reference signal for gravitational wave signal, *y*(*n*) represents the gravitational wave signal after denoising by different algorithms, *L* represents the length of the signal.

It should be noted that in calculating the signal-to-noise ratio, peak signal-to-noise ratio, and mean squared error of the gravitational wave signals, Eqs25-27 were not directly used. Instead, the gravitational wave signals recorded by the H1 and L1 detectors were used as the expected signal and observed noise.

Using different algorithms to calculate the signal-to-noise ratio, peak signal-to-noise ratio, and mean square error of gravitational wave signals quantifies their denoising effects, further highlighting the superiority of the proposed multiscale decomposition denoising algorithm. The results are shown in Tables 2 and 3 respectively.

**Table 2:** Comparison of evaluation metrics for different algorithms applied to GW150914 [5001-9500] segment.

Algorithm	SNR	PSNR	MSE
Kalman filtering	26.4404	35.8858	1.4943e-41
Wavelet soft threshold	15.1942	24.0826	1.9909e-40
Wavelet hard threshold	13.5802	22.1766	2.3870e-40
The proposed algorithm	39.7296	47.7625	1.6930e-43

**Table 3:** Comparison of evaluation metrics for different algorithms applied to GW150914 [32501-37000] segment.

Algorithm	SNR	PSNR	MSE
Kalman filtering	24.7340	32.8471	2.2725e-41
Wavelet soft threshold	18.1787	26.2067	2.0281e-40
Wavelet hard threshold	16.7961	24.3422	2.4135e-40
The proposed algorithm	35.1830	47.2390	2.0410e-43

From the table, it can be seen that the denoising algorithm proposed in this paper achieves the highest SNR and lowest MSE. Upon calculation, the average SNR obtained is 37.4563 and the average MSE is 1.8670*e*^−43^. Furthermore, denoising using Kalman filtering results in an average SNR of 25.5872 and an average MSE of 1.8834*e*^−41^, with slight fluctuations, indicating some denoising effectiveness, albeit with limited improvement, consistent with the visual results in Figs 8 and 9. Denoising with wavelet thresholding shows no significant difference in average SNR, with values of 16.6865 and 15.1881, respectively; similarly, the obtained average MSE values are close, at 2.0095*e*^−40^ and 2.4403*e*^−40^. The algorithm proposed in this paper increases the average SNR by 36.65% and reduces the average MSE by two orders of magnitude. This once again demonstrates the superiority of the multiscale decomposition denoising algorithm proposed in this study.

**Fig 10:**
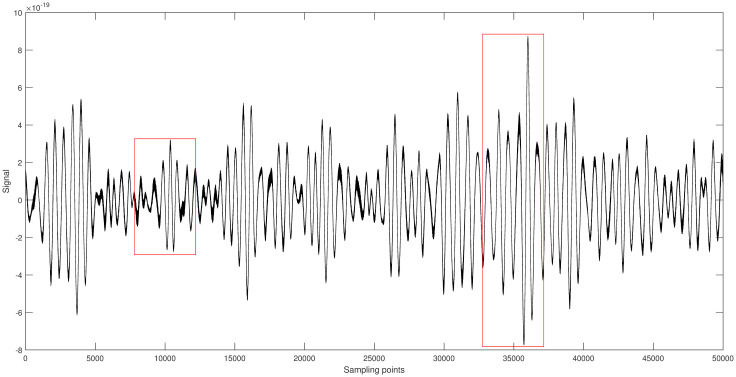
Space gravitational wave signal GW170817 diagram.

**Fig 11:**
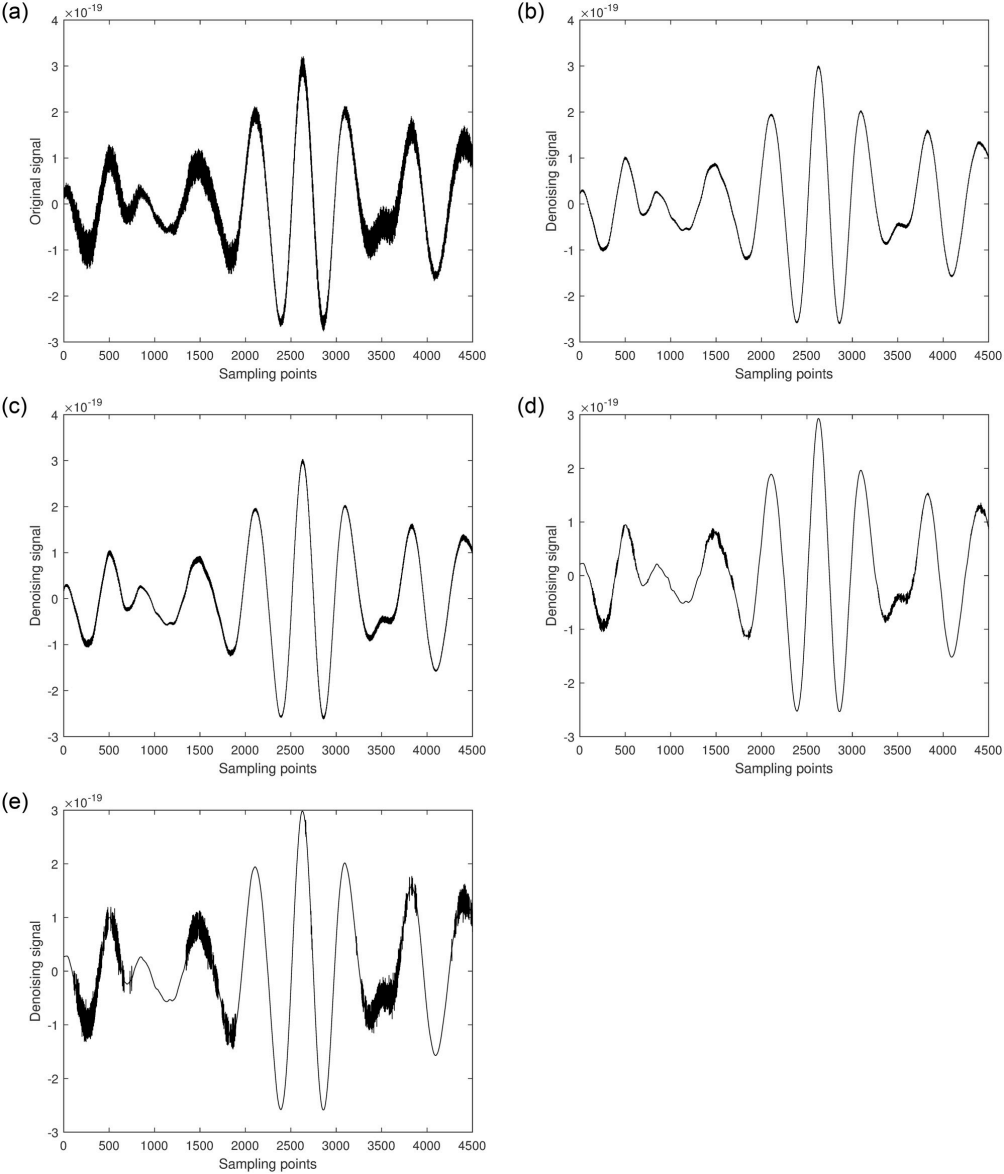
a. [7751-12250] part of the original signal. b. Multiscale decomposition algorithm for [7751-12250] part denoising processing. c. Kalman filter for [7751-12250] part denoising processing. d. Wavelet soft threshold for [7751-12250] part denoising processing. e. Wavelet hard threshold for [7751-12250] part denoising processing.

**Fig 12:**
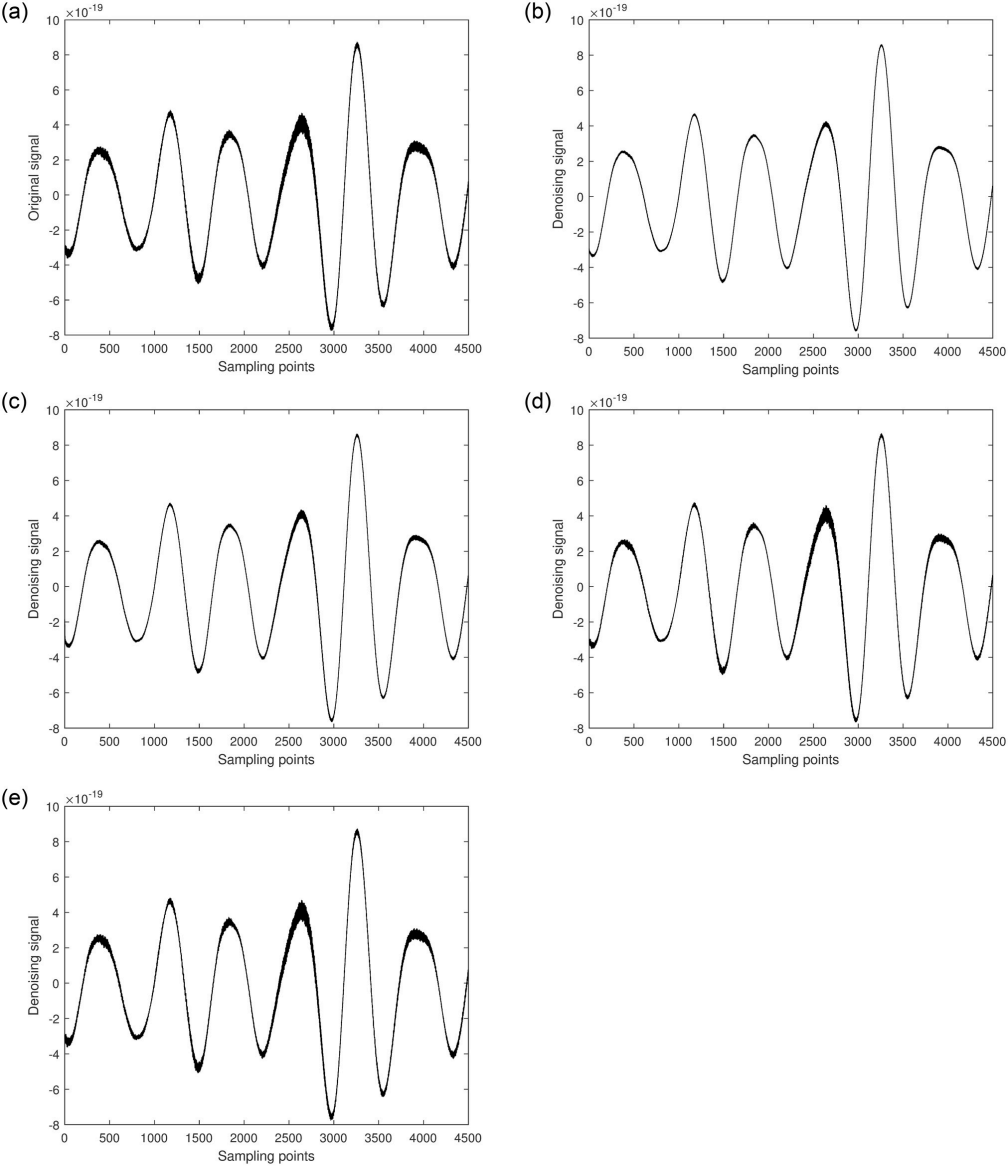
a. [32751-37250] part of the original signal. b. Multiscale decomposition algorithm for [32751-37250] part denoising processing. c. Kalman filter for [32751-37250] part denoising processing. d. Wavelet soft threshold for [32751-37250] part denoising processing. e. Wavelet hard threshold for [32751-37250] part denoising processing.

Next, the denoising process is applied to GW170817. Fig 10 shows the raw signal of GW170817. Two segments of the signal, [7751-12250] and [32751-37250], each with a length of 4500, are randomly selected. Different algorithms are used to denoise each segment, and the results are shown in Figs 11 and 12.

From the figures, it can be observed that compared to the original GW170817 gravitational wave signal, the quality of the segments [7751-12250] and [32751-37250] improved to varying degrees after denoising with Kalman filtering. Although this method can significantly restore the gravitational wave signal, it performs poorly in areas with high local noise frequencies. Similarly, the quality of the segments [7751-12250] and [32751-37250] improved after denoising with wavelet thresholding. Among these, the hard thresholding wavelet method achieved the poorest denoising results, particularly in the noisy segments where effective signal components were barely delineated, and most remained mixed with noise, showing some artifacts. The soft thresholding wavelet method performed better, effectively removing most of the noise and filtering out high-frequency noise, resulting in a smoother waveform. However, some effective local waveforms still showed insignificant denoising improvements. The multiscale decomposition denoising method proposed in this paper achieved the best denoising results. It effectively removed most of the noise from the original gravitational wave signal and accurately delineated almost all effective signal components, resulting in a much smoother waveform. This demonstrates the reliability and superiority of the multiscale decomposition denoising algorithm proposed in this study.

Using different algorithms to calculate the signal-to-noise ratio, peak signal-to-noise ratio, and mean square error of gravitational wave signals quantifies their denoising effects, further highlighting the superiority of the proposed multiscale decomposition denoising algorithm. The results are shown in Tables 4 and 5 respectively.

**Table 4:** Comparison of evaluation metrics for different algorithms applied to GW170817 [7751-12250] segment.

Algorithm	SNR	PSNR	MSE
Kalman filtering	24.6132	37.2392	1.6895e-41
Wavelet soft threshold	19.7989	28.9391	1.3109e-40
Wavelet hard threshold	16.7658	25.1007	2.6361e-40
The proposed algorithm	35.9748	44.7625	1.5398e-43

**Table 5:** Comparison of evaluation metrics for different algorithms applied to GW170817 [32751-37250] segment.

Algorithm	SNR	PSNR	MSE
Kalman filtering	25.6863	31.8116	1.3929e-41
Wavelet soft threshold	20.3634	29.2019	1.1295e-40
Wavelet hard threshold	17.5975	27.8937	2.2381e-40
The proposed algorithm	38.4079	46.5447	1.6212e-43

From the table, it can be seen that the denoising algorithm proposed in this paper achieves the highest SNR and the lowest MSE. The calculated average SNR is 37.1914, and the average MSE is 1.5805*e*^−43^. Additionally, denoising using Kalman filtering results in an average SNR of 25.1498 and an average MSE of 1.5412*e*^−41^, both showing slight variations, indicating some denoising effectiveness but limited improvement, consistent with the results shown in Figs 11 and 12. The wavelet thresholding method shows no significant difference in average SNR, with values of 20.0812 and 17.1817, respectively; the obtained average MSE values are also similar, at 1.2202*e*^−40^ and 2.4371*e*^−40^. The proposed algorithm increases the average SNR by 33.05% and reduces the average MSE by two orders of magnitude. This further demonstrates the superiority of the multiscale decomposition denoising algorithm proposed in this paper.

Due to the fact that GW150914 and GW170817 are two well-known gravitational wave events with strong and distinct signals that can be clearly distinguished in observational data, we selected the gravitational wave event GW170104, which has a relatively lower signal-to-noise ratio, for low-SNR experiments.

**Fig 13:**
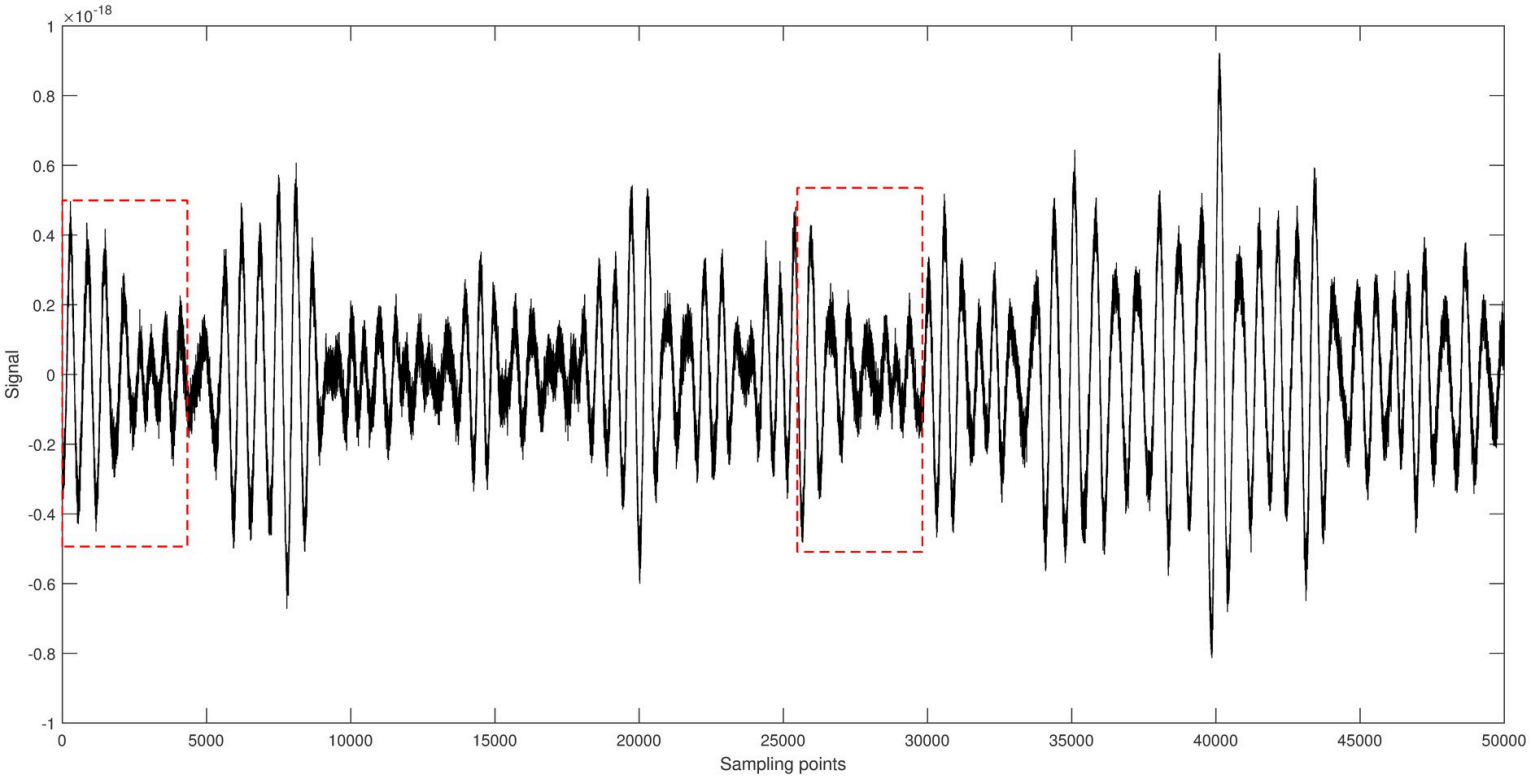
Space gravitational wave signal GW170104 diagram.

**Fig 14:**
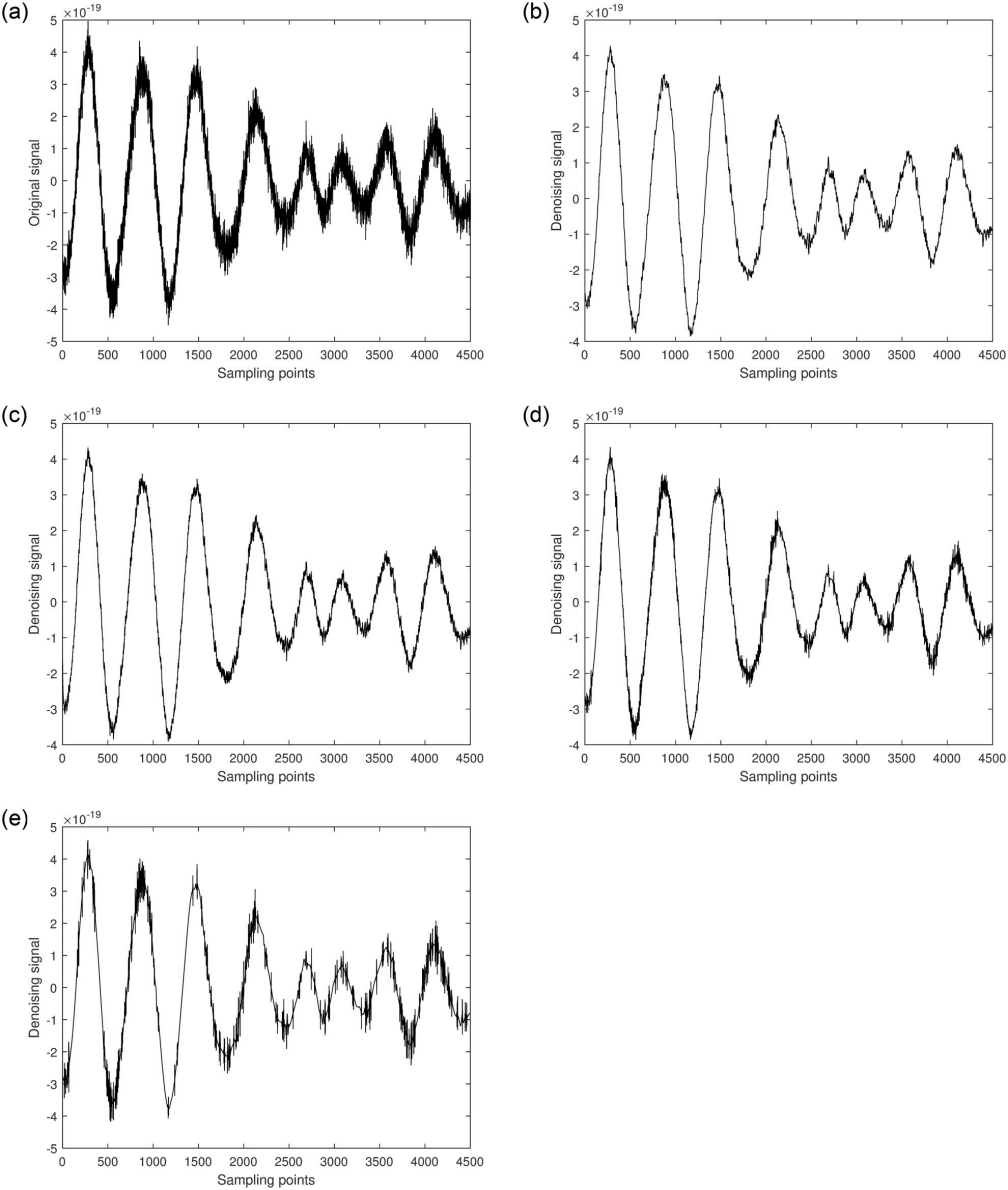
a. [1-4500] part of the original signal. b. Multiscale decomposition algorithm for [1-4500] part denoising processing. c. Kalman filter for [1-4500] part denoising processing. d. Wavelet soft threshold for [1-4500] part denoising processing. e. Wavelet hard threshold for [1-4500] part denoising processing.

**Fig 15:**
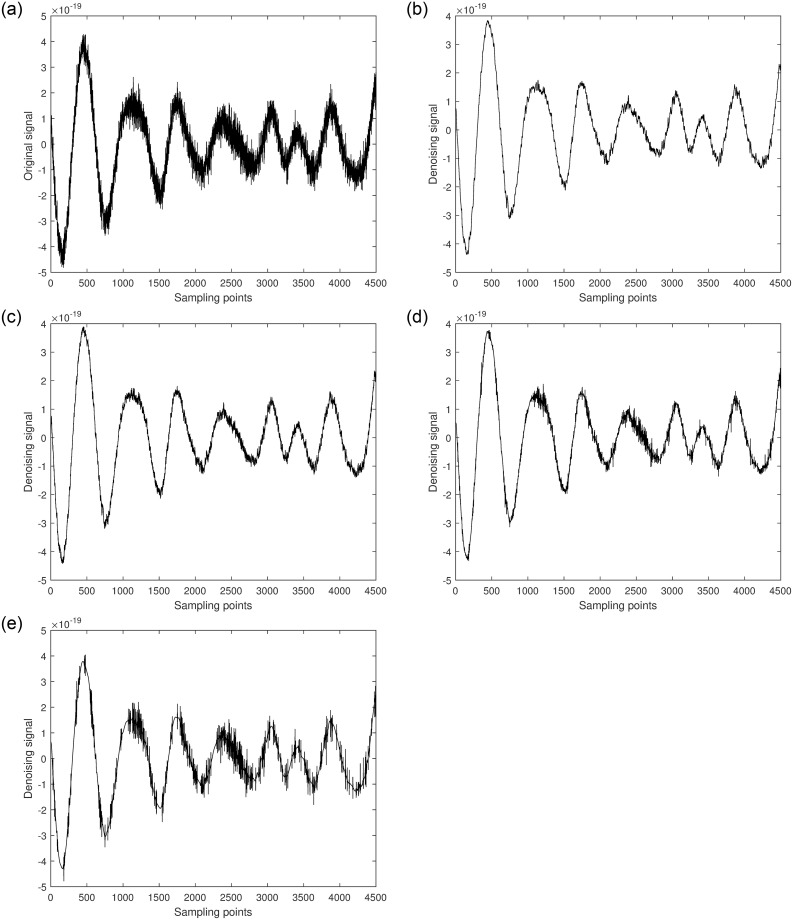
a. [25501-30000] part of the original signal. b. Multiscale decomposition algorithm for [25501-30000] part denoising processing. c. Kalman filter for [25501-30000] part denoising processing. d. Wavelet soft threshold for [25501-30000] part denoising processing. e. Wavelet hard threshold for [25501-30000] part denoising processing.

Next, the denoising process is applied to GW170104. Fig 13 shows the raw signal of GW170104. Two segments of the signal, [1-4500] and [25501-30000], each with a length of 4500, are randomly selected. Different algorithms are used to denoise each segment, and the results are shown in Figs 14 and 15.

From the figures, it can be observed that compared to the original GW170104 gravitational wave signal, the quality of the segments [1-4500] and [25501-30000] improved to varying degrees after denoising with Kalman filtering. Although this method can significantly restore the gravitational wave signal, it performs poorly in areas with high local noise frequencies. Similarly, the quality of the segments [1-4500] and [25501-30000] improved after denoising with wavelet thresholding. Among these, the hard thresholding wavelet method achieved the poorest denoising results, particularly in the noisy segments where effective signal components were barely delineated, and most remained mixed with noise, showing some artifacts. The soft thresholding wavelet method performed better, effectively removing most of the noise and filtering out high-frequency noise, resulting in a smoother waveform. However, some effective local waveforms still showed insignificant denoising improvements. The multiscale decomposition denoising method proposed in this paper achieved the best denoising results. It effectively removed most of the noise from the original gravitational wave signal and accurately delineated almost all effective signal components, resulting in a much smoother waveform. This demonstrates the reliability and superiority of the multiscale decomposition denoising algorithm proposed in this study.

Using different algorithms to calculate the signal-to-noise ratio, peak signal-to-noise ratio, and mean square error of gravitational wave signals quantifies their denoising effects, further highlighting the superiority of the proposed multiscale decomposition denoising algorithm. The results are shown in Tables 6 and 7 respectively.

**Table 6:** Comparison of evaluation metrics for different algorithms applied to GW170104 [1-4500] segment.

Algorithm	SNR	PSNR	MSE
Kalman filtering	19.7817	28.8923	9.9899e-40
Wavelet soft threshold	16.1765	24.1015	7.2957e-39
Wavelet hard threshold	14.9407	20.3406	3.7028e-39
The proposed algorithm	24.3479	32.2929	8.0746e-42

**Table 7:** Comparison of evaluation metrics for different algorithms applied to GW170104 [25501-30000] segment.

Algorithm	SNR	PSNR	MSE
Kalman filtering	19.7114	27.2963	7.2625e-40
Wavelet soft threshold	16.1412	24.2661	6.9913e-39
Wavelet hard threshold	13.8767	22.4028	1.5782e-39
The proposed algorithm	23.3348	31.8864	7.5424e-42

From the table, it can be seen that the denoising algorithm proposed in this paper achieves the highest SNR and the lowest MSE. The calculated average SNR is 23.8414, and the average MSE is 7.8085*e*^−42^. Additionally, denoising using Kalman filtering results in an average SNR of 19.7466 and an average MSE of 8.6262*e*^−40^, both showing slight variations, indicating some denoising effectiveness but limited improvement, consistent with the results shown in Figs 14 and 15. The wavelet thresholding method shows no significant difference in average SNR, with values of 16.1589 and 14.4087, respectively; the obtained average MSE values are also similar, at 7.1435*e*^−39^ and 2.6405*e*^−39^. The proposed algorithm increases the average SNR by 22.24% and reduces the average MSE by two orders of magnitude. This further demonstrates the superiority of the multiscale decomposition denoising algorithm proposed in this paper.

**Fig 16:**
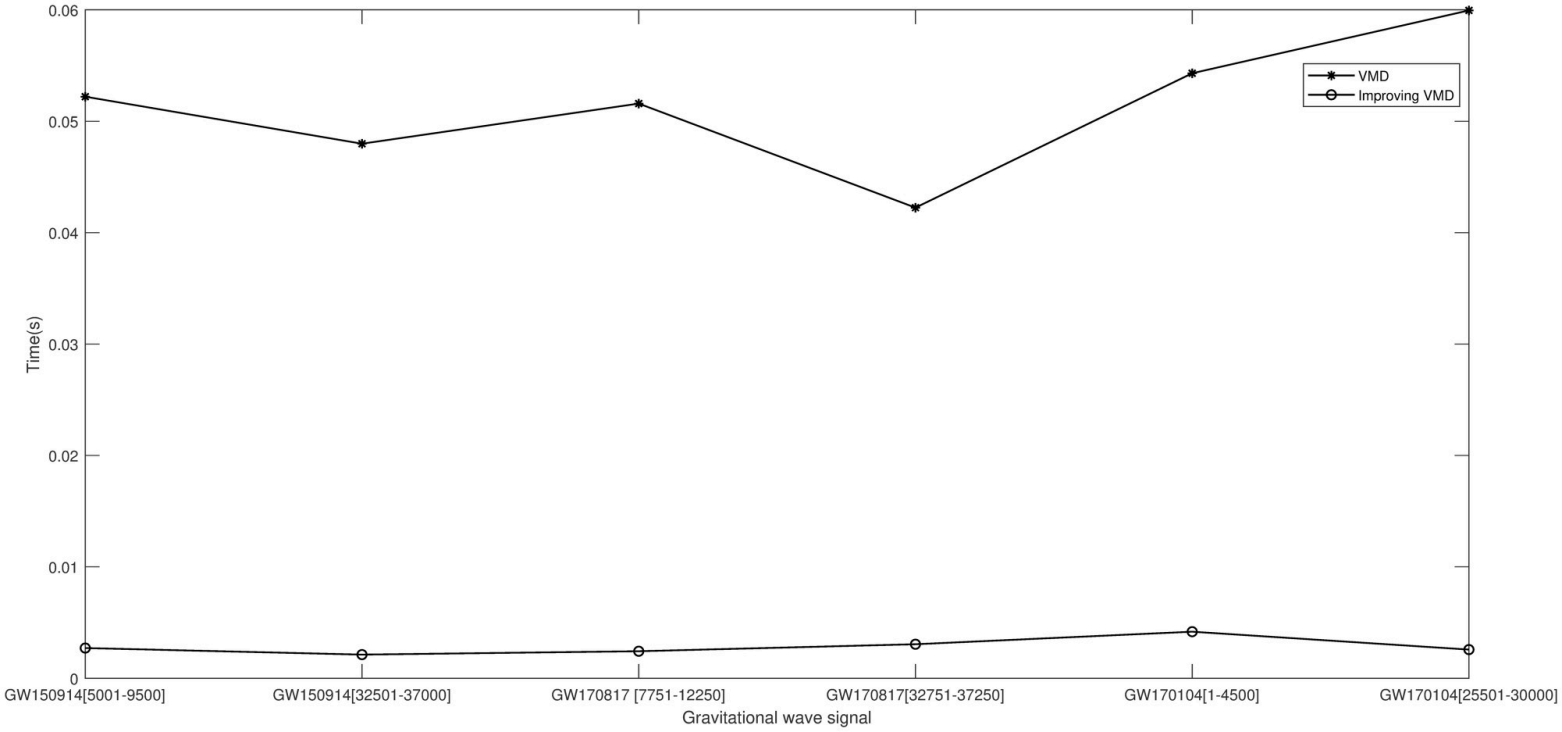
Convergence speed of the improved algorithm.

The momentum gradient-based variational mode optimization algorithm enhances the convergence speed. This is demonstrated by processing and comparing two segments of the GW150914 signal, [5001-9500] and [32501-37000], two segments of the GW170817 signal, [7751-12250] and [32751-37250], and two segments of the GW170104 signal, [1-4500] and [25501-30000]. The convergence results are shown in Fig 16.

As clearly shown in the figure, the momentum gradient-based variational mode decomposition optimization algorithm improves the convergence speed compared to the original variational mode decomposition algorithm, accelerates response time, and enhances the least mean square algorithm’s adaptability to noise removal, demonstrating the superiority of the improved variational mode decomposition algorithm.

## Conclusion

This paper proposes a denoising method for space-based gravitational wave signals based on multiscale decomposition with momentum gradient descent, addressing the requirements for noise reduction in space gravitational wave detection signals. The study conducts an in-depth investigation into the denoising of gravitational wave signals. In order to mitigate the challenge of low signal-to-noise ratio resulting from the faint nature of gravitational wave signals and their susceptibility to noise, this study employs the variational mode decomposition algorithm and the least mean squares algorithm in the context of space-based gravitational wave detection. This approach aims to remove noise from gravitational wave signals at smaller scales. In order to address the challenges of slow convergence and the existence of multiple local optima in the variational mode decomposition algorithm, this study incorporates momentum gradient descent and multiscale concepts to optimize the variational mode decomposition algorithm, thereby enhancing its convergence speed. Comprehensive experiments and both qualitative and quantitative results demonstrate that the proposed algorithm significantly outperforms other algorithms, achieving an average signal-to-noise ratio improvement of 30.65% and an average mean squared error improvement by two orders of magnitude from *e*^−39^ to *e*^−42^. These findings underscore the algorithm’s effectiveness and superiority in noise suppression and enhanced detection capability, effectively meeting the noise suppression requirements for space-based gravitational wave detection.
